# Synthesis under high pressure: crystal structure and properties of cubic Dy_36_O_11_F_50_[AsO_3_]_12_ ∙ H_2_O

**DOI:** 10.3389/fchem.2024.1354690

**Published:** 2024-03-26

**Authors:** Felix Christian Goerigk, Ralf Jules Christian Locke, Thomas Schleid

**Affiliations:** Faculty of Chemistry, Institute for Inorganic Chemistry, University of Stuttgart, Stuttgart, Germany

**Keywords:** high-pressure synthesis, oxoarsenates(III), crystal structures, crystal water, dysprosium, oxide fluorides, X-ray diffraction, microprobe analysis

## Abstract

The multi-anionic compound with the composition Dy_36_O_11_F_50_[AsO_3_]_12_ ∙ H_2_O, which can be described in the non-centrosymmetric cubic space group *F* $\bar{4}3$ *c*, already shows an unusually large unit cell with an axis of *a* = 2587.59(14) pm. Its crystal structure exhibits isolated ψ^1^-tetrahedral [AsO_3_]^3–^ anions, but both the coordination numbers and the linking schemes of the Dy^3+^-centered polyhedra differ significantly from the mostly layered structures described so far in literature. (Dy1)^3+^ is sevenfold coordinated by oxygen atoms and F^−^ anions, forming a capped trigonal prism [(Dy1)O_4.333_F_2.667_]^8.333–^, and the remaining two cations (Dy2)^3+^ and (Dy3)^3+^ both reside in an eightfold coordination of anions. In both cases they form slightly distorted square antiprisms, which have the compositions of [(Dy2)O_3.667_F_4.333_]^8.667–^ and [(Dy3)O_4.667_F_3.333_]^9.667–^, respectively. Some of the oxygen atoms are not part of ψ^1^-[AsO_3_]^3–^ tetrahedra, but occur as O^2–^ anions and one of these even shares a common crystallographic position with fluoride (F^−^). It is also worth mentioning that the single crystals were obtained as comparatively large cubes with an edge length of several 100 µm providing very good data with regard to single-crystal X-ray diffraction. To verify the simultaneous presence of oxygen and fluorine, electron-beam microprobe analysis was carried out, and a single-crystal Raman spectrum ruled out the presence of hydroxide anions or protonated [AsO_3_]^3–^ groups, but proved the interstitial crystal-water molecules, which could not be determined precisely by the crystal-structure refinement.

## Introduction

Regardless of the oxidation state at the involved arsenic atoms, rare-earth metal oxoarsenates can serve well as either host materials or concentrated phosphors for lumogenious applications. This holds for the *monazite*-, *xenotime*-, and *scheelite*-type oxoarsenates(V) *RE*[AsO_4_] (Schäfer and Will, 1971; Lohmüller et al., 1973; Long and Stager, 1977; Schäfer et al., 1979; Brahim et al., 2002; Kang et al., 2005a; Kang, 2009; Kang and Schleid, 2005; Schmidt et al., 2005; Hartenbach et al., 2006; Golbs et al., 2009; Metzger, 2012; Metzger et al., 2016; Ledderboge et al., 2018; Goerigk, 2021; Adala et al., 2022) (*RE* = rare-earth metal: Sc, Y, La, Ce–Lu), where O^2−^-to-As^5+^ ligand-to-metal charge-transfer processes (LMCT) within the tetrahedral [AsO_4_]^3−^ anions have a beneficial impact on the necessary energy transfer as well as for the oxoarsenates(III) *RE*[AsO_3_] and *RE*_4_[As_2_O_5_]_2_[As_4_O_8_] (Ben Hamida et al., 2005; Kang, 2009; Kang and Schleid, 2006; Ben Hamida, 2007; Metzger, 2012; Metzger et al., 2012; Ledderboge et al., 2014; Ledderboge, 2016; Locke et al., 2023), where the O^2−^-to-As^3+^ LMCT is supported by the lone-pair antenna at the trivalent arsenic centers. The latter occur as ψ^1^-tetrahedral [AsO_3_]^3−^ groups either isolated in the first cases (Pb[SeO_3_]- or K[ClO_3_]-type *RE*[AsO_3_]) or vertex-condensed to di- and tetranuclear anions (*pyro*-[As_2_O_5_]^4−^ and *cyclo-*[As_4_O_8_]^4−^) for the latter ones (*RE*_4_[As_2_O_5_]_2_[As_4_O_8_] ≡ 2 × *RE*_2_As_4_O_9_). Driven by the influence of fluxing halides during the corresponding preparation efforts, halide-derivatized rare-earth metal(III) oxoarsenates(III) were obtained for the first time, exhibiting the empirical formula *RE*_5_ *X*_3_[AsO_3_]_4_ (*X* = F (Ledderboge and Schleid, 2014; Ledderboge, 2016; Goerigk, 2021), Cl (Kang, 2009; Hamida and Wickleder, 2006; Ben Hamida, 2007; Schander, 2009; Ledderboge, 2016; Goerigk et al., 2019), and Br (Ledderboge, 2016; Goerigk, 2021)). With *X* = Cl and Br as soft halide anions, they occur as layered structures, while the fluoride-derivatives represent three-dimensionally hard materials according to the Pearson HSAB concept of “hard and soft acids and bases“ (Pearson, 1963). For this reason, they are well-suited as host substrates, which secure energy transfer with minimal losses from the rigid lattice and its hard components (*RE* ^3+^, F^−^ and [AsO_3_]^3−^) to the *Ln* ^3+^ activator cations, such as Eu^3+^ and Tb^3+^ as most prominent ones. The crystallization and single-phase preparation of most fluoride oxoarsenates(III) *RE*_5_F_3_[AsO_3_]_4_ was not an easy task in the past, so we tried different synthetic pathways to tackle this challenge. In the case of Dy_5_F_3_[AsO_3_]_4_, one of these attempts involved a droplet of water added to the appropriate mixture of the well-ground solid starting materials (Dy_2_O_3_, DyF_3_, and As_2_O_3_ in a 2 : 1: 2 molar ratio) in order to heat it in a sealed gold ampoule within the set-up of a Boyd and England-type piston-cylinder high-pressure apparatus. As a surprising result, big well-faceted single crystals of what turned out to be Dy_36_O_11_F_50_[AsO_3_]_12_ with one molecule of interstitial water per formula unit were obtained. We report here on its fascinating unique crystal structure and several analytic methods to confirm its true nature as dysprosium(III) oxide fluoride oxoarsenate(III) hydrate according to Dy_36_O_11_F_50_[AsO_3_]_12_ · H_2_O.

## Experimental

**FIGURE 1:**
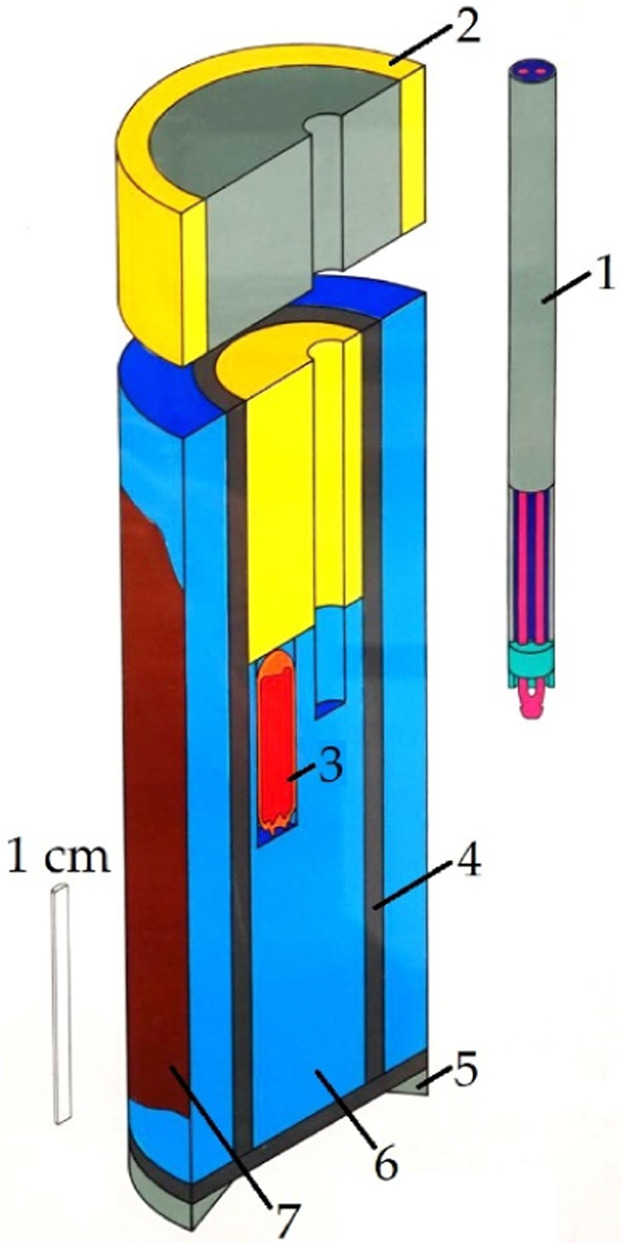
Schematic structure of the rock salt-type pressure cell of the Boyd and England piston-cylinder apparatus 1) sheathed thermocouple (NiCr-Ni), 2) upper seal (fired pryophyllite ring and steel plug), 3) gold capsule with sample material (4 mm outer diameter), 4) steel furnace, 5) lower piston ring, 6) rock-salt cell, and 7) copper paste (Boyd and England, 1960; Massonne and Schreyer, 1986).

**FIGURE 2:**
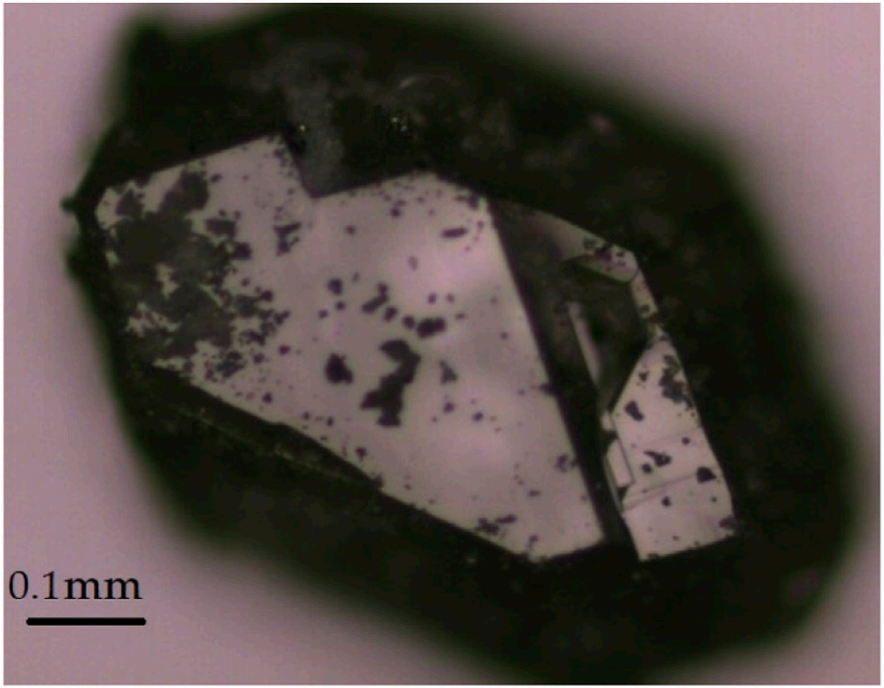
A colorless transparent single crystal of Dy_36_O_11_F_50_[AsO_3_]_12_ ∙ H_2_O.

Since phase-pure and single-crystalline *RE*_5_F_3_[AsO_3_]_4_ representatives are relatively difficult to access and preparative attempts to obtain them with conventional techniques from fused silica ampoules often led to oxosilicates, a reaction under high-pressure conditions in an inert gold capsule was considered as an alternative. Dysprosium sesquioxide (Dy_2_O_3_), dysprosium trifluoride (DyF_3_), and arsenic sesquioxide (As_2_O_3_) served as reactants in a molar ratio of 2 (164 mg): 1 (48 mg): 1 (87 mg). A fine blend of the reactants was prepared and filled into a gold ampoule (4 mm diameter and 10 mm length), which already contained some demineralized water (30 µL). To prevent water loss while sealing the ampoule, the upper fold was closed by cold welding with a pressure of almost 10 tons at the fold. The ampoule produced this way was placed in a rock-salt pressure cell (Figure 1) and then inserted into the pressure tube of the end-loaded high-pressure piston-cylinder reactor (Boyd and England-type) (Boyd and England, 1960; Massonne and Schreyer, 1986). The operating pressure was set to just 8.5 kbar at a temperature of 500°C for 4 days, then lowered to 400°C and dwelled for a further 3 days. After the end of the experiment and opening of the ampoule, a few very large cube-shaped colorless transparent single crystals, some with an edge length of several 100 µm (Figure 2), were found. These did not cause any rotation of the linearly polarized light in transmitted light under crossed polarizers, which is why the presence of a crystal in the cubic crystal system was assumed (Nickel, 1971). Due to the size, the habit, and the apparently cubic symmetry, it was initially wrongly assumed that the crystals should be sodium chloride (NaCl) deriving from the pressure cell. However, as the crystals did not dissolve in water, one of them was isolated and examined using single-crystal X-ray diffraction. The measurement was carried out with a STADI-VARI single-crystal diffractometer (Stoe & Cie, Darmstadt, Germany). A face-centered cubic metric with *a* ≈ 2590 pm was found, which could not be assigned to any known class of rare-earth metal(III) compounds. The composition Dy_36_O_11_F_50_[AsO_3_]_12_ ∙ H_2_O in space group *F* $\bar{4}$ 3*c* was determined using direct methods and the structure was refined with the SHELX-97 (Sheldrick, 1997; Herrendorf et al., 1999; Sheldrick, 2008) program package. Based on the selected reactants and their initial weights, however, a synthesis without secondary phases was not possible, as the stoichiometric coefficients for the used weights of the reactants did not allow this. The powder X-ray diffraction pattern of Dy_36_O_11_F_50_[AsO_3_]_12_ ∙ H_2_O was recorded using a STADI-P diffractometer (Stoe & Cie, Darmstadt, Germany) with Ge(222)-monochromatized copper radiation (*λ* = 154.06 pm). The sample material was measured in transmission geometry in order to minimize the influence of texture effects and to receive an acceptable signal-to-noise-ratio. About 50 mg of the product was slightly ground and transferred on an amorphous adhesive film (scotch magic tape). For investigations using the Bragg–Brentano geometry, there was not enough sample material available (only about 150 mg product). In the measurement setup, a Stoe position-sensitive detector (PSD) with an angular resolution of 0.073° was employed. As step size, 0.02° in the area from 5° to 70° (2*θ*) was chosen. A Raman spectrum for the single crystal (excitation wavelength: *λ* = 638 nm) measured with a Raman microscope (XploRa, Horiba, Kyoto) can be found as well as electron-beam microprobe measurements (SX-100, Cameca, Gennevilliers) in the corresponding subsections.

## Results and discussion

### Crystal-structure description

**FIGURE 3:**
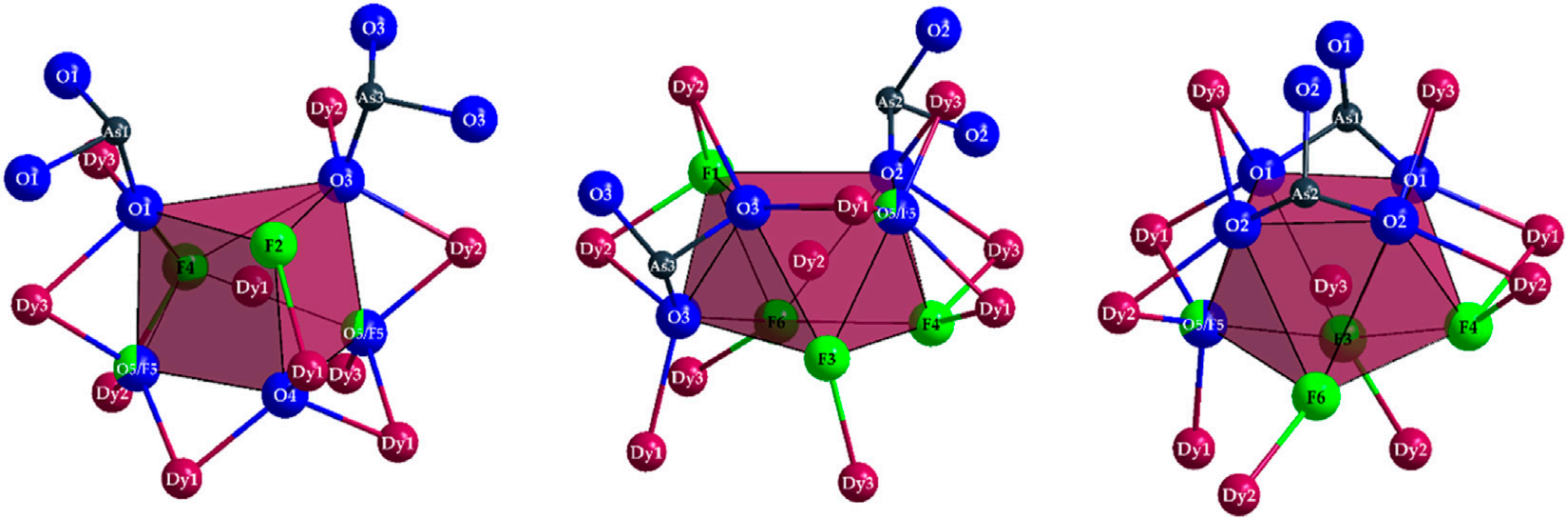
Coordination polyhedra of the compositions [(Dy1)O_4.333_F_2.667_]^8.333–^ (*left*), [(Dy2)O_3.667_F_4.333_]^8.667–^ (*mid*), and [(Dy3)O_4.667_F_3.333_]^9.667–^ (*right*) as well as the As^3+^ cations covalently bonded to most oxygen atoms and their remaining oxygen atoms in ψ^1^-tetrahedral [AsO_3_]^3–^ anions in the crystal structure of Dy_36_O_11_F_50_[AsO_3_]_12_ ∙ H_2_O.

The cubic compound Dy_36_O_11_F_50_[AsO_3_]_12_ ∙ H_2_O crystallizes in the non-centrosymmetric space group *F*4 $\bar{3}$ *c* with *a* = 2587.59(14) pm and eight formula units in the unit cell (Table 1). Three crystallographically unique sites are present for the Dy^3+^ cations, all of which reside at general Wyckoff sites 96*h* (Table 2). (Dy1)^3+^ is sevenfold coordinated by oxygen atoms and F^−^ anions, forming a capped trigonal prism [(Dy1)O_4.333_F_2.667_]^8.333–^ (Figure 3, *left*). The “odd" stoichiometric coefficients are due to the common O5/F5 position, which has to be statistically occupied to ^2^/_3_ by oxygen and to ^1^/_3_ by fluorine for the overall stoichiometry to remain charge neutral, unless further mixed occupancies are to be introduced on other anion sites. Here, dysprosium-oxygen bonds with lengths of 225–244 pm and distances of the (Dy1)^3+^ cation to the F^−^anions of 231–244 pm occur (Table 3). The two cations (Dy2)^3+^ and (Dy3)^3+^ have eightfold coordination spheres by anions, in both cases forming slightly distorted square antiprisms, which show compositions of [(Dy2)O_3.667_F_4.333_]^8.667–^ and [(Dy3)O_4.667_F_3.333_]^9.667–^ (Figure 3, *mid* and *right*). The interatomic distances from the Dy^3+^ cations to the anions occurring here are in quite similar ranges with values of *d*(Dy2–O) = 224–252 pm, *d*(Dy2–F) = 224–245 pm and *d*(Dy3–O) = 227–250 pm, *d*(Dy3–F) = 224–235 pm, although by increasing the coordination number from seven to eight, a slight increase for the maximum distance can be observed.

**TABLE 1:** Crystallographic data of Dy_36_O_11_F_50_[AsO_3_]_12_ ∙ H_2_O and their determination.

Formula	Dy_36_O_11_F_50_[AsO_3_]_12_ ∙ H_2_O
Crystal system	cubic
Space group	*F* $\bar{4}$ 3*c* (no. 219)
Lattice parameter, *a*/pm	2587.59(14)
Number of formula units, *Z*	8
Calculated density, *D*_x_/g ∙ cm^–3^	6.492
Molar volume, *V*_m_/cm^3^ ∙ mol^–1^	1304.523
Measurement limit, 2*θ*/°	60.96
Measurement range, ±*h*_max_ = ±*k*_max_ = ±*l*_max_	36
Electron sum, *F*(000)/e^–^	28848
Absorption coefficient, *µ*/mm^–1^	35.332
Diffractometer	Stoe Stadi-Vari
Analytical radiation	Mo-*K*_α_ (*λ* = 71.07 pm)
Measured reflections	200828
Symmetrically independent ones	2221
*R*_int_/*R*_σ_	0.092/0.034
*R*_1_/*R*_1_ with |*F*_o_| ≥ 4*σ*(*F*_o_)	0.044/0.029
w*R*_2_/*GooF*	0.061/1.017
Flack X parameter	–0,01(3)
Residual electron density, *ρ*_max/min_/e^–^ ∙ 10^−6^ pm^–3^	1.58/–1.71
CSD number	2310780

**TABLE 2:** Fractional atomic coordinates, *Wyckoff* positions, and coefficients of the equivalent isotropic displacement parameters of Dy_36_O_11_F_50_[AsO_3_]_12_ ∙ H_2_O.

Atom	Site	*x*/*a*	*y*/*b*	*z*/*c*	*U*_eq_/pm^2^
Dy1	96*h*	0.203007(16)	0.001157(16)	0.072966(16)	183.4(9)
Dy2	96*h*	0.294678(16)	0.084582(15)	0.145213(16)	158.3(9)
Dy3	96*h*	0.190631(16)	0.148864(16)	0.072279(15)	155.0(9)
As1	32*e*	0.08211(3)	0.08211(3)	0.08211(3)	158(3)
As2	32*e*	0.19160(3)	0.19160(3)	0.19160(3)	153(3)
As3	32*e*	0.40950(4)	0.40950(4)	0.40950(4)	174(3)
O1	96*h*	0.0666(2)	0.0984(2)	0.1467(2)	174(12)
O2	96*h*	0.2152(2)	0.1345(2)	0.1612(2)	153(12)
O3	96*h*	0.3458(3)	0.1094(3)	0.0710(3)	208(13)
O4	24*d*	^1^/_4_	0	0	193(25)
O5*	96*h*	0.2476(2)	0.0822(2)	0.0725(2)	169(11)
F1	32*e*	0.3341(2)	0.3341(2)	0.3341(2)	324(23)
F2	48*f*	0.1432(4)	0	0	475(26)
F3	96*h*	0.3137(3)	0.0065(3)	0.1130(3)	348(15)
F4	96*h*	0.2220(2)	0.0261(2)	0.1618(2)	301(14)
F5**	96*h*	0.2476(2)	0.0822(2)	0.0725(2)	169(11)
F6	96*h*	0.2668(2)	0.1969(2)	0.0734(2)	306(14)
Ow	8*a*	0	0	0	1557(243)
H***	32*e*	0.0215	0.0215	0.0215	–

* *s.o.p.* = ^2^/_3_, ** *s.o.p.* = ^1^/_3_ for the mixed *s*ite-*o*ccupation *p*robability; *** *s.o.p.* = ^1^/_2_.

**TABLE 3:** Selected interatomic distances (*d*/pm) in Dy_36_O_11_F_50_[AsO_3_]_12_ ∙ H_2_O.

Contact	Multiplicity	Distance/pm	Contact	Multiplicity	Distance/pm
**[(Dy1)O_4.333_F_2.667_]^8.333–^ polyhedra**			**[(Dy3)O_4.667_F_3.333_]^9.667–^ polyhedra**		
Dy1–O4	1×	224.6(4)	Dy3–F3	1×	224.3(6)
Dy1–O5/F5	1×	230.7(6)	Dy3–O5/F5	1×	226.9(5)
Dy1–O1	1×	232.8(6)	Dy3–F6	1×	232.9(5)
Dy1–O5'/F5′	1×	239.2(6)	Dy3–F4	1×	235.9(6)
Dy1–F4	1×	243.8(6)	Dy3–O1	1×	239.2(6)
Dy1–F2	1×	244.2(7)	Dy3–O2	1×	241.6(6)
Dy1–O3	1×	244.3(7)	Dy3–O2′	1×	247.2(6)
**[(Dy2)O**_**3.667**_ **F**_**4.333**_ **]** ^**8.667–**^ **polyhedra**			Dy3–O1′	1×	250.6(7)
Dy2–F3	1×	224.0(6)	**[AsO**_**3**_ **]** ^**3–**^ **ψ** ^**1**^ **-tetrahedra**		
Dy2–O5/F5	1×	224.4(6)	As1–O1	3×	177.0(6)
Dy2–F6	1×	230.6(6)	As2–O2	3×	178.2(6)
Dy2–F1	1×	239.8(4)	As3–O3	3×	179.2(7)
Dy2–O3	1×	241.9(7)	**H**_**2**_ **O molecule (crystal water)**		
Dy2–F4	1×	245.3(7)	Ow–H	2×	96.4
Dy2–O2	1×	246.4(6)			
Dy2–O3′	1×	251.7(6)			

In the binary dysprosium sesquioxide (Dy_2_O_3_, *bixbyite* type), the dysprosium-oxygen distances are in the range of 215–254 pm, while in dysprosium oxide fluoride (DyOF, YOF type), they occur in a narrower interval of 227–234 pm. Thus, the refined bond lengths in Dy_36_O_11_F_50_[AsO_3_]_12_ ∙ H_2_O are in good agreement with those of the simpler representatives Dy_2_O_3_ and DyOF (Rudenko and Boganov, 1970; Dutton et al., 2012). For the distances to the F^−^ anions, a similar situation is observed, as they are 242–249 pm in DyOF and in binary DyF_3_ (YF_3_ type) in between 234 and 274 pm (Garashina and Sobolev, 1971; Dutton et al., 2012). Here, F^−^ anions always show slightly longer bonds to the Dy^3+^ cations as compared to the O^2–^ anions, while the O^2–^ and F^−^ anions as well as arsenic-bonded oxygen atoms in Dy_36_O_11_F_50_[AsO_3_]_12_ ∙ H_2_O have quite similar distances to the Dy^3+^ cations (Table 3).

**FIGURE 4:**
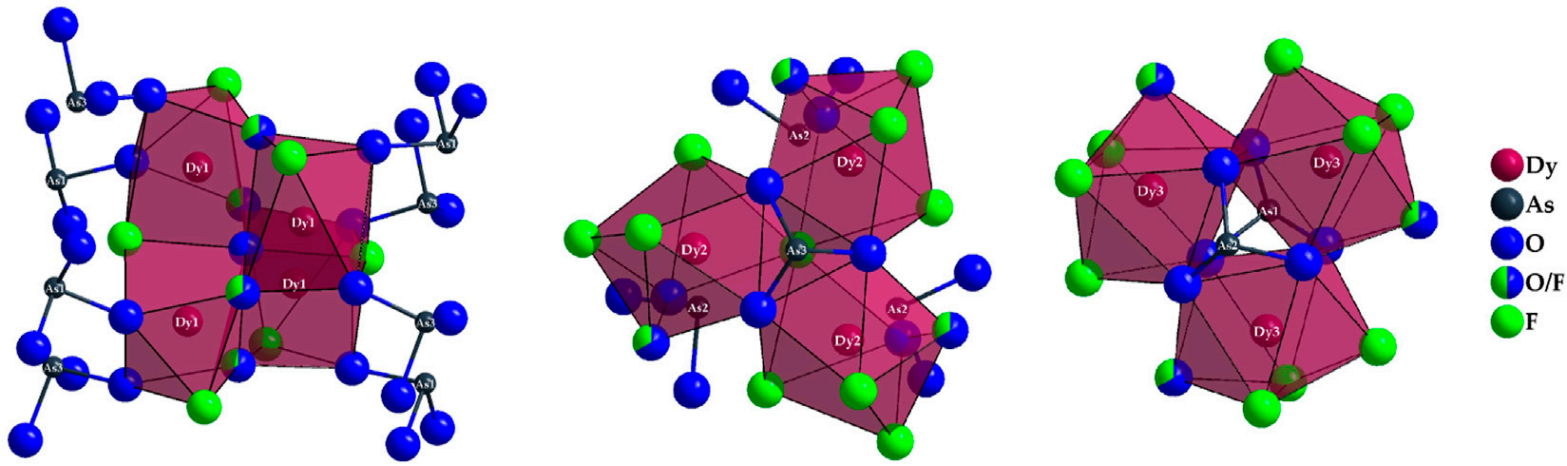
Linkage pattern of Dy^3+^-centered coordination polyhedra in the crystal structure of Dy_36_O_11_F_50_[AsO_3_]_12_ ∙ H_2_O with drawing of the linkage to polyhedra with the crystallographically identical central cations (Dy1)^3+^ (*left*), (Dy2)^3+^ (*mid*), and (Dy3)^3+^ (*right*).

The polyhedra around the Dy^3+^ cations are linked by both corners and edges with different patterns. For a better understanding, in the following, first the linkages of Dy^3+^-centered polyhedra to each other with crystallographically identical Dy^3+^ cations are described. The [(Dy1)O_4.333_F_2.667_]^8.333–^ polyhedra are connected to each other by common edges, and always four of these polyhedra form a tetrahedral body in which the central oxide anion (O4)^2–^ is tetrahedrally surrounded exclusively by four (Dy1)^3+^ cations (Figure 4, *left*). Two oxoarsenate(III) units [AsO_3_]^3–^ are attached to each polyhedron, with only one common oxygen atom being present. In the case of the [(Dy2)O_3.667_F_4.333_]^8.667–^ antiprisms, the situation is different: here, only three polyhedra are linked to each other exclusively by common edges. It is interesting to note that all three polyhedra share the (F1)- anion and the three oxygen atoms of an oxoarsenate(III) unit of the (As2)^3+^ cation provide the remaining edge links.

The [(As2)O_3_]^3–^ anion thus bridges all three (Dy2)^3+^-centered polyhedra. This creates a distorted tetrahedral gap at the center of an imaginary triangular surface through the three (Dy2)^3+^ cations (Figure 4, *mid*). In the case of the [(Dy3)O_4.667_F_3.333_]^9.667–^ polyhedra, the situation is quite similar. Here, three antiprisms are connected by common edges, but the main difference to the case of the (Dy2)^3+^ cation lies in the bridging atoms. Here, the linked [(Dy3)O_4.667_F_3.333_]^9.667–^ polyhedra are surrounded on both sides by ψ^1^-tetrahedra [(As3)O_3_]^3–^, forming an empty trigonal prism from the oxygen atoms of these oxoarsenate(III) units (Figure 4, *right*).

The other linkage patterns are quite similar and are described here, but not further shown graphically. The polyhedra around (Dy1)^3+^ are connected with three (Dy2)^3+^-centered polyhedra each, with one edge and two corner links present. There are also three contacts to the (Dy3)^3+^-centered polyhedra, with two edge and only one corner linkage occurring here. For the [(Dy2)O_3.667_F_4.333_]^8.667–^ antiprism there are, in addition to the already mentioned, three contacts to the capped [(Dy1)O_4.333_F_2.667_]^8.333–^ prisms (twice via edge and once via corner); there are also contacts to four (Dy3)^3+^-centered polyhedra, with two edge and two corner-connections. The [(Dy3)O_4.667_F_3.333_]^9.667–^ antiprism is linked to three (Dy1)^3+^- and four (Dy2)^3+^- centered polyhedra, where it is linked to the [(Dy1)O_4.333_F_2.667_]^8.333–^ polyhedra twice via edge and once over corner and to the [(Dy2)O_3.667_F_4.333_]^8.667–^ antiprisms twice via edge and twice via corner.

**FIGURE 5:**
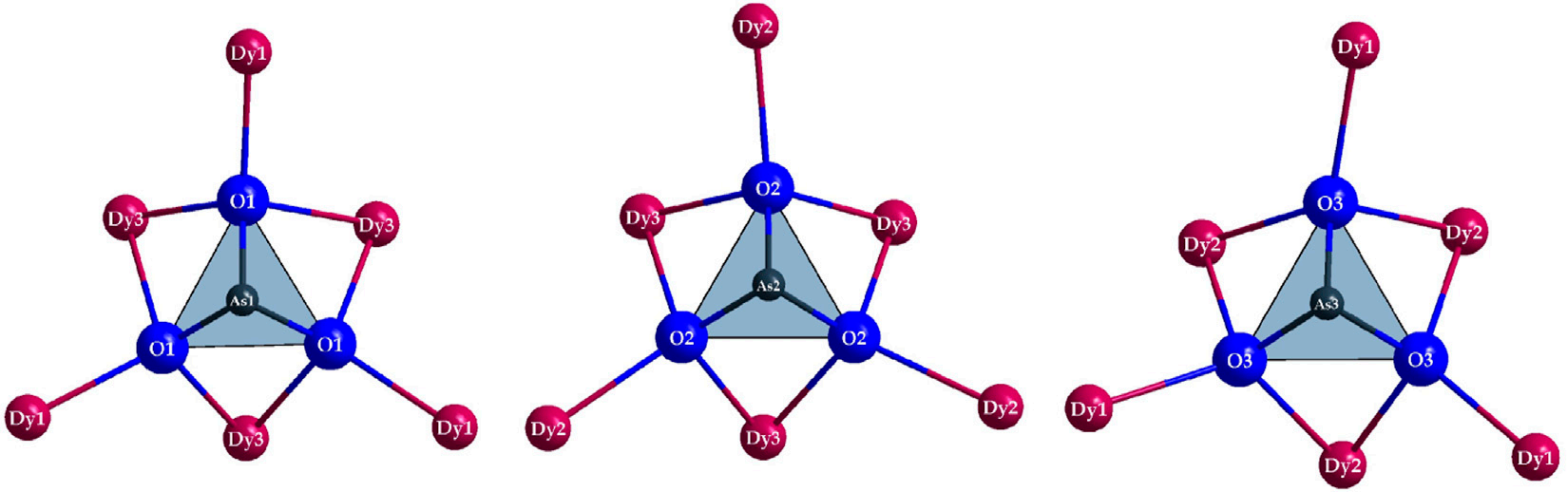
The first and second coordination sphere of the As^3+^ cations occurring in the crystal structure of Dy_36_O_11_F_50_[AsO_3_]_12_ ∙ H_2_O as ψ^1^-tetrahedral [AsO_3_]^3–^ anions with their Dy^3+^ decoration.

The crystal structure exhibits three crystallographically different sites for the As^3+^ cations as well (Table 2). The first and second coordination spheres of these arsenic centers are identical in all three cases, only the bond lengths show a slight variance. In all cases, isolated ψ^1^-tetrahedra [AsO_3_]^3–^ are formed, whose oxygen atoms each have contact with a terminal and two bridging Dy^3+^ cations (Figure 5). Thereby, the arsenic-oxygen distances with values of 177–179 pm are in a rather narrow range, but very close to the typical arsenic(III)-oxygen bonds in *claudetite*-I (172–181 pm) (Pertlik, 1978a), *claudetite*-II (177–182 pm) (Pertlik, 1975), and *arsenolite* (179 pm, 3×; Table 3) (Pertlik, 1978b), to name just those of the crystalline As_2_O_3_ modifications.

**FIGURE 6:**
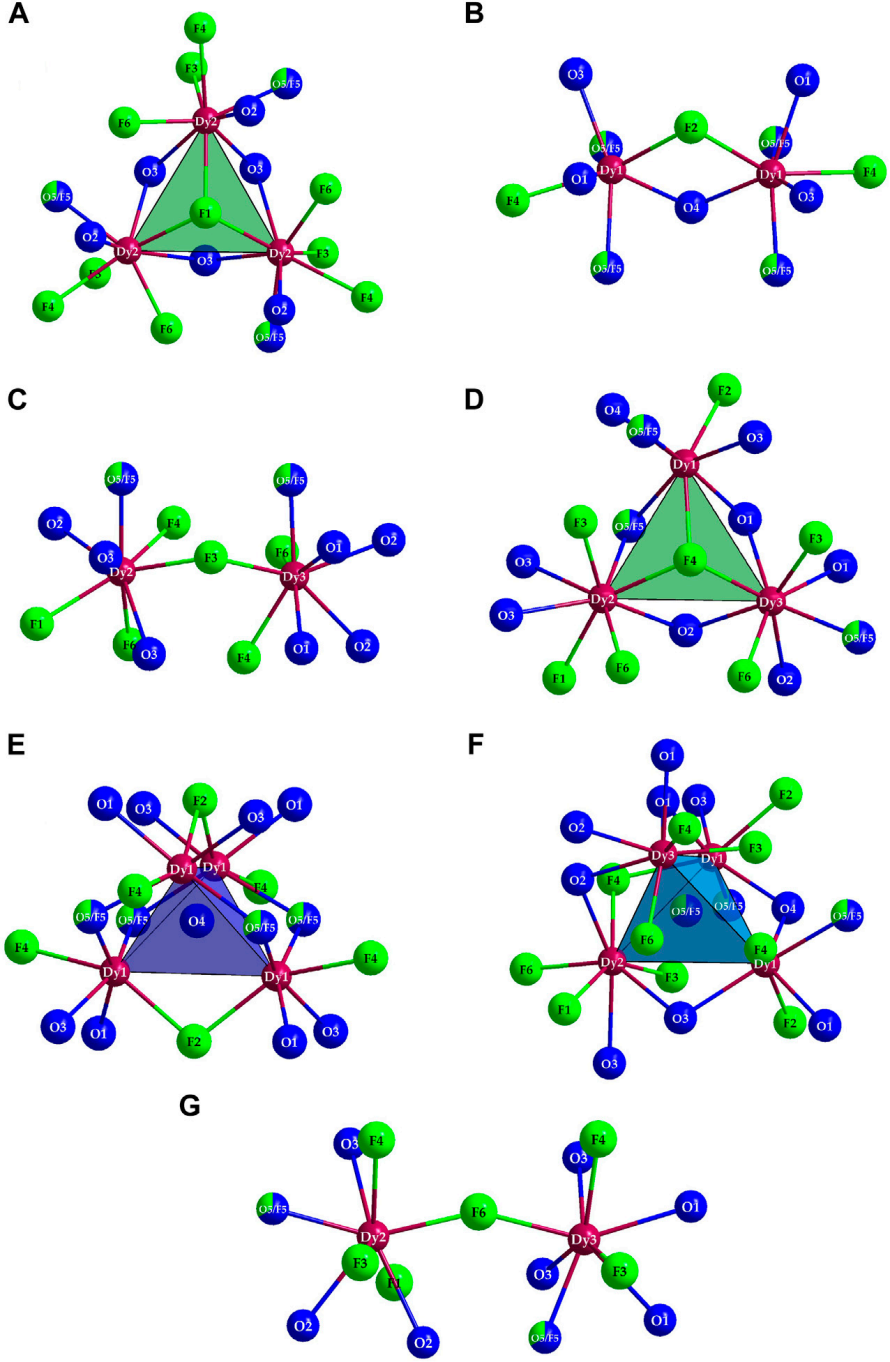
The anion sites occurring in the crystal structure of Dy_36_O_11_F_50_[AsO_3_]_12_ ∙ H_2_O without binding contacts to As^3+^ cations and their extended environments for (F1)^−^ **(A)**, (F2)^−^ **(B)**, (F3)^−^ **(C)**, (F4)^−^ **(D)**, (O4)^2−^ **(E)**, (O5/F5)1.667^−^ **(F)** and (F6)^−^ **(G)**.

In the crystal structure, there are also eight anion positions, which do not maintain any contacts to the As^3+^ cations (Figure 6). The corresponding elements were assigned to the sites in such a way that all oxygen atoms are tetrahedrally surrounded by Dy^3+^ cations (*C.N.* = 4), while angled and trigonal coordination spheres also occur for the F^−^ anions (*C.N.* = 2 and 3). Two peculiarities stand out here: on the one hand, there is a mixed-occupied position with the (O5)^2–^ and (F5)^–^ anions, which is necessary for the charge neutrality of the compound; on the other hand, it is not possible to say with certainty whether only this site is actually mixed and all the other anion positions of Table 2 are occupied by only one kind of non-metallic element.

The (O4)^2–^-centered (Dy^3+^)_4_ tetrahedron could also be mixed with fluoride, since the coordination spheres hardly differ. However, since the counts (= multiplicity of *Wyckoff* positions) of the (O4)^2–^ and (O5)^2–^ sites and the possible variance of the negative charges due to the different *Wyckoff* positions (24*d* versus 96*h*) differ, charge neutrality would not be achievable by a pure mixed occupation of the (O4)^2–^ site. From a merely mathematical point of view, it would also be possible for both positions to be mixed, but for the sake of simplicity, only a mixed occupation of the (O5)^2–^ site was assumed as a structural model. Furthermore, oxygen as an O^2–^ anion also strives for higher coordination numbers than F^−^ anions, which is why a mixed occupation of the only two- and three-coordinated anion sites can be regarded as rather unlikely. The tendency of oxygen atoms, which are not covalently bonded to As^3+^ cations, to be coordinated by *Ln* ^3+^ cations in quaternary lanthanoid(III) oxide halide oxoarsenates(III) in the form of [O*Ln*_4_]^10+^ tetrahedra is already well-known from literature. In the compounds with the structured formula *Ln*_3_O*X*[AsO_3_]_2_ (*Ln* = La–Nd, Sm–Dy for *X* = Cl, *Ln* = La–Nd, Sm, Gd–Dy for *X* = Br, and *Ln* = Pr for *X* = I) such [O*Ln*_4_]^10+^ tetrahedra are present, which share common edges according to $\begin{array}{c} 1\\ \infty \end{array}$ {[O*Ln* $\begin{array}{c} e\\ 4/2 \end{array}$ ]^4+^} (*e* = edge-connecting) to infinite chains along their tetragonal *c*-axis (Kang et al., 2005b; Kang, 2009; Kang and Schleid, 2007; Ben Yahia et al., 2009; BenYahia et al., 2010; Ledderboge, 2016).

**FIGURE 7:**
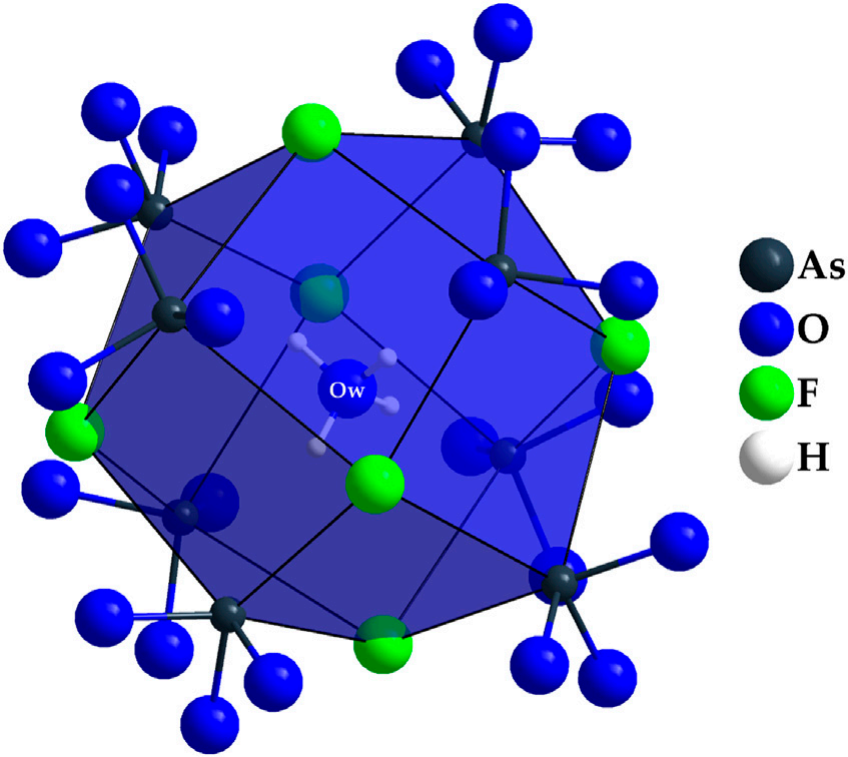
Tetrahedral coordination environment of the oxygen atom Ow in the crystal structure of Dy_36_O_11_F_50_[AsO_3_]_12_ ∙ H_2_O, which can be explained by an intercalated crystal-water molecule in a rhomboid-dodecahedral cavity formed by (As1)^3+^, (As2)^3+^, and (F2)^–^.

**FIGURE 8:**
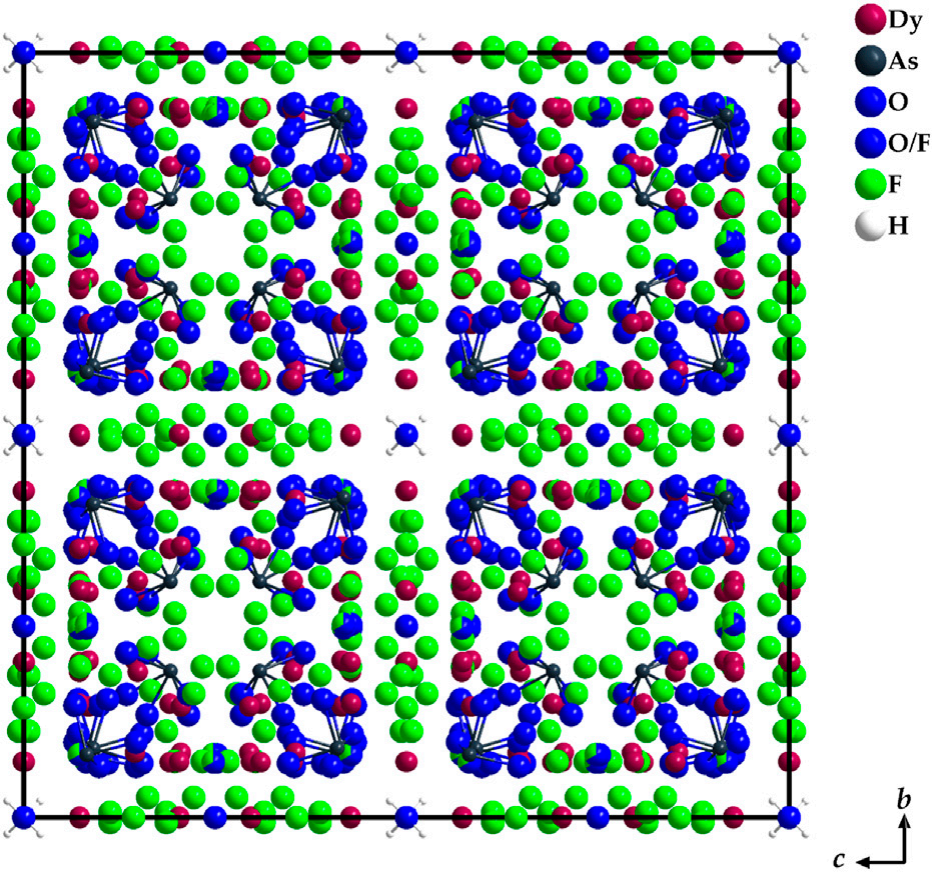
Section of the crystal structure of Dy_36_O_11_F_50_[AsO_3_]_12_ ∙ H_2_O as viewed along the *a*-axis. The covalent bonds of the As^3+^ cations to their oxygen atoms within the ψ^1^-tetrahedral [AsO_3_]^3–^ anions and the ones in the crystal-water molecules are emphasized.

The point of highest residual electron density is located at the origin of the unit cell (8*a*: 0, 0, 0). In terms of the scattering power, the intensity corresponds to a position occupied by an oxygen atom. However, this hypothetical oxygen atom has no binding contacts with other particles present in the unit cell. The closest contact is at 368 pm to As^3+^ cations and at 371 pm to F^−^ anions, well outside the range of plausible chemical bonding. However, based on the synthesis parameters, one explanation would be that this could be the oxygen atom of a crystal water molecule. Upon further search for a suitable hydrogen atom, residual electron density can also be found at a distance of about 96.4 pm (32*e*: *x*, *x*, *x*, with *x* = 0.0215) from this oxygen atom. However, due to the applicable symmetry operations, this hydrogen atom would be arranged tetrahedral around the central oxygen atom, a circumstance that does not seem to make sense from a structural-chemical point of view. An under-occupation of this hydrogen position by one half to achieve a neutral water molecule (H_2_O) would be possible and very likely with a H–O–H angle of 109.5°, but hardly be detected by X-ray diffraction. The hypothetical empirical formula of the title compound would then be Dy_36_O_11_F_50_[AsO_3_]_12_ ∙ H_2_O. The coordination environment of this interstitial crystal-water molecule is shown in Figure 7 with minimal H∙∙∙As distances of 272 pm and H∙∙∙F distances of 325 pm, both far too long for significant bridging hydrogen bonds. Figure 8 presents a section of the whole crystal structure including the cell edges.

### Powder X-Ray Diffraction

**FIGURE 9:**
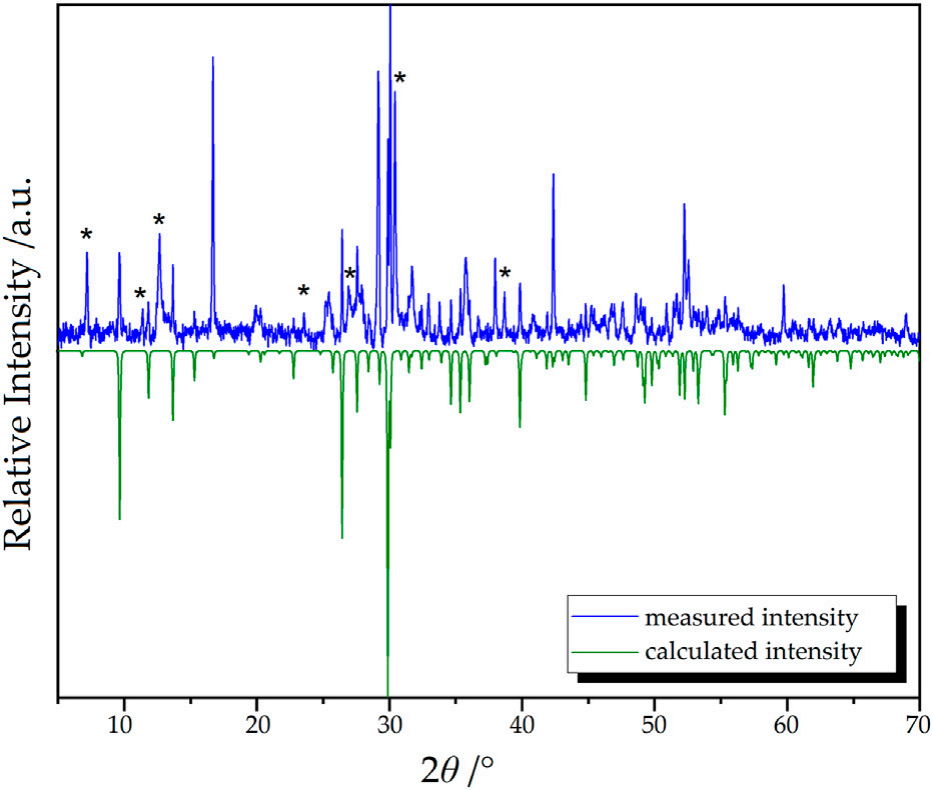
Measured (blue) and calculated (green) powder X-ray diffractogram of Dy_36_O_11_F_50_[AsO_3_]_12_ ∙ H_2_O. Unindexed reflections are marked with asterisks.

In order to investigate the composition of the obtained material, powder X-ray diffraction techniques were applied (Figure 9). Since the available amount of the sample was rather low, the signal-to-noise-ratio of the data is challenging.

The target compound Dy_36_O_11_F_50_[AsO_3_]_12_ ∙ H_2_O could be identified as the main phase in the mixture. However, multiple reflections that do not belong to the target compound are also present (marked with “asterisks" *). Regarding the reaction conditions, the theoretical patterns of the starting materials and possible by-products were used to check if one or several of these phases are present in the reaction mixture. Nevertheless, none of the tested compounds (As_2_O_3_ in its *claudetite*- and *arsenolite*-type, respectively, Dy_2_O_3_ (*A*-, *B*- and *C*-type), DyOF, DyF_3_, Dy[AsO_4_] (*xenotime*- and *scheelite*-type), and even Dy_5_F_3_[AsO_3_]_4_) could be identified as possible side-phases.

Since no As_2_O_3_ and Dy_2_O_3_ seem to be residual, a complete reaction of the starting materials should have taken place that lead to multiple products of which the target compound Dy_36_O_11_F_50_[AsO_3_]_12_ ∙ H_2_O can be seen as the main phase.

### Raman spectroscopy

**FIGURE 10:**
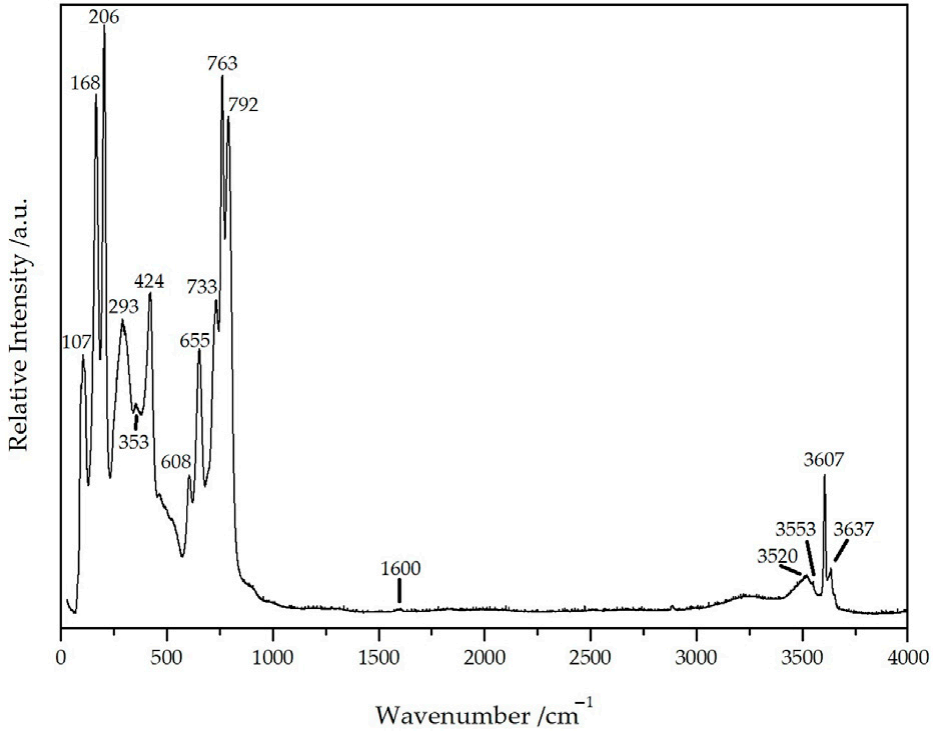
Single-crystal Raman spectrum of Dy_36_O_11_F_50_[AsO_3_]_12_ ∙ H_2_O recorded at an excitation wavelength of *λ* = 638 nm.

To further investigate the mysterious crystal and to verify the interstitial crystal-water molecules, a single-crystal Raman spectrum of Dy_36_O_11_F_50_[AsO_3_]_12_ ∙ H_2_O was recorded with an excitation wavelength of *λ* = 638 nm (Figure 10). The peaks in the range from 100 to 500 cm^–1^ (107, 168, 206, 293, 353, 424 cm^–1^) can be assigned to the stretching vibrations *ν*(Dy(O,F)) and the deformation vibrations *δ*(AsO_3_) with the three strongest ones probably belonging to *ν*(Dy(O,F)) and lattice vibrations. The two peaks at 608 and 655 cm^–1^ with the shoulder at 700 cm^–1^ are caused by the antisymmetric stretching vibrations *ν*_as_(AsO_3_) of the three crystallographically different [AsO_3_]^3–^ anions, whereas the significantly stronger three peaks at 733, 763, and 792 cm^–1^ undoubtedly belong to the symmetric stretching vibrations *ν*_s_(AsO_3_) of these units. The slight elevation at 1600 cm^–1^ can be assigned to the deformation vibrations *δ*(H_2_O) of the crystal-water molecules, while the very sharp peak at 3607 cm^1^ belongs to the symmetrical valence vibration *ν*_s_(H_2_O) of them and the somewhat smaller one at 3637 cm^–1^ stems from the antisymmetrical one *ν*_as_(H_2_O). The two weaker and broader peaks at 3520 and 3553 cm^–1^ can be interpreted as results from the stretching vibrations *ν*(H_2_O)_*n*_ of other very few water species that are bound to the detected crystal-water molecule, but may also well be surface-bonded water of the investigated crystal. The very broad band from 3000 to 3400 cm^–1^ can be attributed to air humidity and is always observable in spectra from this kind of instrument (Weidlein et al., 1981; Weidlein et al., 1986).

### Microprobe analysis

**FIGURE 11:**
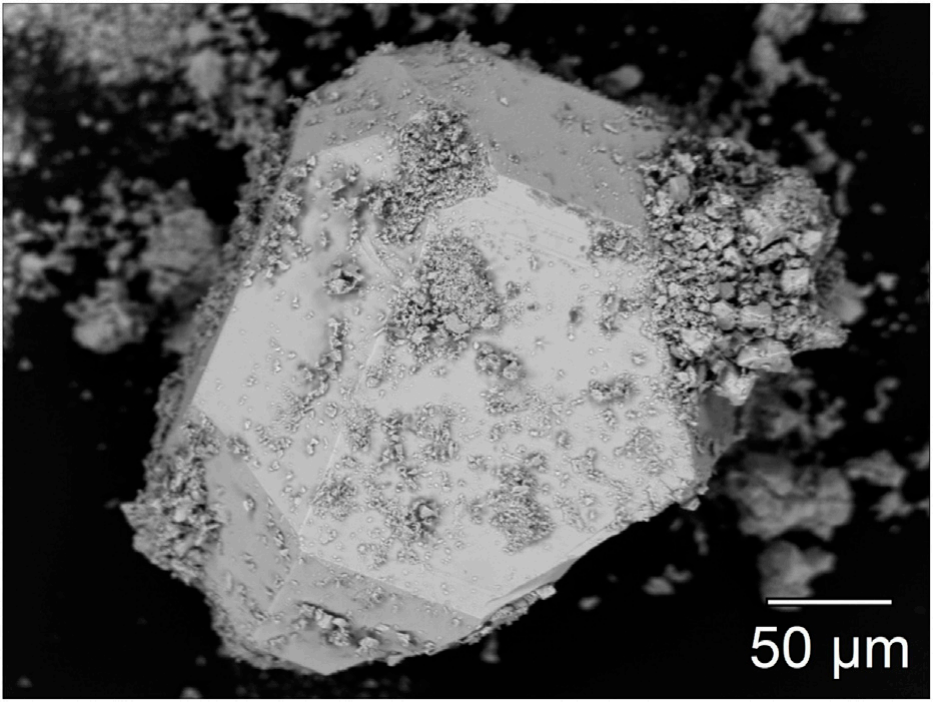
Backscattered electron image of a single crystal of Dy_36_O_11_F_50_[AsO_3_]_12_ ∙ H_2_O recorded at an accelerating voltage of 15 kV and a current of 4 nA. The formed edges of the crystal are clearly visible.

Electron microscopy and X-ray spectroscopy methods were used to further characterize Dy_36_O_11_F_50_[AsO_3_]_12_ ∙ H_2_O. Figure 11 shows a backscattered electron image of a single crystal, which demonstrates the above-average crystal size and the almost isotropic crystal growth. Before the single-crystal X-ray diffraction, the likewise recognizable covering particles were rinsed off by washing them in paraffin oil.

**FIGURE 12:**
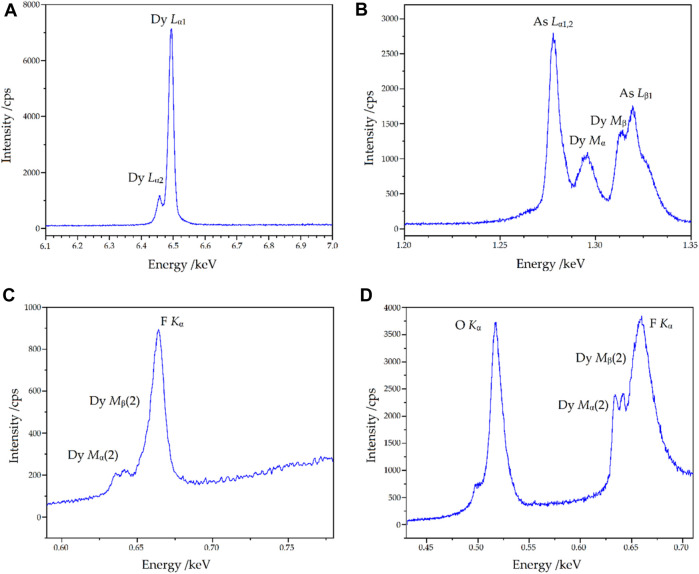
Wavelength-dispersive X-ray spectra (WDXS) of Dy_36_O_11_F_50_[AsO_3_]_12_ ∙ H_2_O in different energy ranges with the relevant emission lines depicted. Given are the regions of Dy-*L*_α_ [LiF crystal, **(A)**], As-*L*_α_ and As-*L*_β_ [TAP crystal, **(B)**], as well as O-*K*_α_ and F-*K*_α_ (on two different spectrometers with multilayer elements, **(C, D)**, respectively).

Qualitative wavelength-dispersive X-ray spectra (WDXS) were recorded to verify the elements assumed in the single-crystal structure refinement. For the lighter elements, pseudo-crystal multilayer elements were used as diffraction crystals in the spectrometers in order to reach the low-energy regions. The relevant spectra of the measurements carried out on the single crystal are shown in Figure 12. The energy range for the heavy atoms dysprosium and arsenic corresponds to the expectations. In the energy ranges not shown here outside these characteristic lines, there are no extraneous bands from other types of atoms. It can also be seen that it would not be possible to detect arsenic using the (energy-dispersive) EDXS method, as interferences of Dy-*M*_α_ and Dy-*M*_β_ with the As-*L*_α_ and As-*L*_β_ lines occur, which can be resolved in the wavelength-dispersive system. The following findings can be obtained in the low-energy range of Figures 12C, D: it can be seen that both fluorine and oxygen are present and, furthermore, higher orders of the Dy-*M*_α_ and Dy-*M*_β_ lines are also detectable in the fluorine region, but with sufficiently low interference, at least for qualitative detection.

## Conclusion

An unexpected cubic dysprosium(III) oxide fluoride oxoarsenate(III) hydrate with the composition Dy_36_O_11_F_50_[AsO_3_]_12_ · H_2_O could be obtained by water-assisted high-pressure synthesis from cold-welded gold ampoules in an attempt to synthesize Dy_5_F_3_[AsO_3_]_4_. Its crystal structure features Dy^3+^ cations with coordination numbers of seven and eight with respect to the non-metal elements (O and F) along with discrete ψ^1^-tetrahedral [AsO_3_]^3−^ anions. Interstitial crystal-water molecules are trapped within a large cavity confined by eight arsenic atoms and six fluoride anions. Upon heating some crystals up to 500 °C for several days, they were destroyed owing to decrepitation under water-release. From a distance, the “odd” composition Dy_36_O_11_F_50_[AsO_3_]_12_ · H_2_O with *a* = 2587.59(14) pm for *Z* = 8 could well be misinterpreted as “Dy_3_OF_4_[AsO_3_] · ^1^/_12_ H_2_O” with *Z* = 96 and even the low water-content might have been overlooked. Under these circumstances, the resulting formula “Dy_36_O_12_F_48_[AsO_3_]_12_ · H_2_O” for *Z* = 8 would have only 12 + 48 = 60 non-metal elements without bonds to arsenic instead of 11 + 50 = 61 as in the true composition. But after all, we have found no evidence for an under-occupation concerning any of these seven non-metal positions in Table 2.

## Data Availability

The original contributions presented in the study are included in the article/Supplementary material, further inquiries can be directed to the corresponding author.
